# Outcomes of Ex-PRESS and Trabeculectomy in a Glaucoma Population of African Origin: One Year Results

**DOI:** 10.5005/jp-journals-10028-1221

**Published:** 2017-08-05

**Authors:** Youssef Dib Bustros, Robert Fechtner, Albert S Khouri

**Affiliations:** 1 Resident, Department of Ophthalmology, Saint George Hospital University Medical Center, Beirut, Lebanon; 2 Professor and Chairman, Department of Ophthalmology, SUNY Upstate Medical University, Syracuse, New York, USA; 3 Associate Professor, Department of Ophthalmology, Rutgers New Jersey Medical School, New Jersey, USA

**Keywords:** African American, Ex-PRESS, Glaucoma, Retrospective study, Trabeculectomy.

## Abstract

**Aim:**

To compare the efficacy and safety of Ex-PRESS glaucoma filtration surgery to trabeculectomy in African origin patients.

**Materials and methods:**

A retrospective chart review was performed on 56 African American patients undergoing Ex-PRESS glaucoma shunt (E) or trabeculectomy (T) between 2004 and 2012. Data collected included intraocular pressure (IOP) and glaucoma medication use at baseline and postoperative week 1, Month (M) 1, M3, M6, M12. Postoperative interventions including laser suture lysis (LSL) and 5FU injections were analyzed. Complete and qualified success rate, and eyes failing and requiring more surgery were determined. Means, SD, chi-square, and Student’s t-test were performed.

**Results:**

A total of 56 subjects (E 28, T 28) were included in the analysis. There was a statistically significant reduction (p < 0.05) in IOP and number of glaucoma medications at all time points compared to baseline for both groups. Extent of IOP reduction between groups was not statistically significant at any time point, except postoperative week 1. Mean number of glaucoma medications between groups was not significant, except at 3 months (lower in EXP group). The cumulative number of postoperative interventions within 3 months (LSL and 5-FU) was significantly greater for the TRAB than EXP (3.89 ± 2.4 *vs* 2.36 ± 2.2, p = 0.007). Success rates were comparable between both groups (Table 2).

**Conclusion:**

Our study is unique in patients of African origin showing statistical significance in the requirement of less postoperative 5-FU injections during the first 3 months following surgery in the Ex-PRESS group *vs* the trabeculectomy group. The Ex-PRESS shunt was as effective as trabeculectomy in reducing IOP and use of glaucoma medications up to 1 year.

**Clinical significance:**

The possible benefit of this article is to help guiding ophthalmologists to select the type of glaucoma filtration surgery to undergo in an African American patient with glaucoma.

**How to cite this article:**

Bustros YD, Fechtner R, Khouri AS. Outcomes of Ex-PRESS and Trabeculectomy in a Glaucoma Population of African Origin: One Year Results. J Curr Glaucoma Pract 2017;11(2):42-47.

## INTRODUCTION

Glaucoma is a disease of the optic nerve that can cause permanent visual loss.^1^ A recent systematic review and metanalysis estimated the current number of people with glaucoma to be 64.3 million and is expected to increase to 76.0 million in 2020 and 111.8 million in 2040.^2^ Glaucoma has a younger age at onset in black patients. In addition, the prevalence, the resistance to treatment, and the aggressivity of the course of this disease seem to be higher in black than white patients.^3,4^

Depending on the severity of glaucoma, surgical interventions may be necessary to lower intraocular pressure (IOP) to a level, i.e., thought to slow the progression of the disease. Trabeculectomy (T), which is a guarded filtration procedure whereby a fistula is created to drain the aqueous humor from the anterior chamber, remains the gold standard surgical method for reduction of IOP in glaucoma patients.^5,6^ A recent study done by Jampel et al showed complete success in 39% of cases and qualified success in 79%.^7^ Trabeculectomy is associated with multiple postoperative complications, such as hyphema, hypotony, choroidal detachment, suprachoroidal hemorrhage, and others.^8^ The development of new techniques and devices in glaucoma surgery aiming to achieve the desired lower levels of IOP with less complications remains important. The Ex-PRESS (E) shunt, which was approved by the Food and Drug Administration (FDA) in 2002, was developed as an alternative to trabeculectomy with the intention of decreasing postoperative complica-tions.^8^ The Ex-PRESS shunt is a stainless steel device, i.e., implanted in the eye to drain the aqueous humor under a scleral flap forming a filtering bleb.

To date, all retrospective and prospective studies comparing Ex-PRESS to trabeculectomy do not include a specific population or target a certain race. To our knowledge, the literature showed a lack of studies comparing the two types of surgeries in patients of African origin who are known to exhibit severe progressive glaucoma and in whom filtering surgery is adversely affected by a robust healing response. Our objective is to compare outcomes of the Ex-PRESS shunt to trabeculectomy in African origin patients over a 1-year follow-up period.

## MATERIALS AND METHODS

A search for all patients undergoing filtering surgery between August 2004 and October 2012 at our institution was conducted. The inclusion criteria included patients diagnosed with open-angle glaucoma >18 years old, undergoing Ex-PRESS glaucoma shunt or trabeculectomy augmented with mitomycin C who completed 12 months of follow-up and who self-reported as being of African origin, including African American and Caribbean patients. This search yielded 56 patients that were then reviewed and included 28 black patients undergoing Ex-PRESS glaucoma shunt and 28 undergoing trabecu-lectomy. All surgery was performed at one center by two Board-certified experienced glaucoma surgeons, and both procedures were augmented with mitomycin C. Exclusion criteria included African origin patients who lacked 1-year follow-up or who had secondary glaucoma (trauma, uveitic, neovascular, other). Patients with previous incisional glaucoma surgery, penetrating keratoplasty or other incisional anterior segment surgery besides phacoemulsification were not included. The protocol for this study was approved by the Institutional Review Board of the university.

Trabeculectomy was performed in standard technique. For both procedures, a corneal traction suture was placed, if needed, to rotate the eye inferiorly, then a limbal incision was created with Westcott scissors to create a fornix based flap. The incision was about 5 mm in length. Tenon and all subconjunctival scarring was dissected with a Blumenthal dissector. Mitomycin C 0.4 mg/mL soaked unto two semicircular corneal shield pieces (diameter 7 mm) were then placed under the conjunctiva for about 2 minutes and then removed and the area copiously irrigated with balanced salt solution. Wet field cautery was then performed to achieve hemostasis. A diamond blade preset at 300 microns was used to delineate a 3 × 3 mm scleral flap that was then dissected with a 57 no. blade. A paracentesis was performed temporally to allow access to the anterior chamber and reform it with balanced salt solution during the case. For trabeculectomy a Kelly punch was used to create a sclerotomy under the scleral flap followed by a peripheral iridectomy. In the eyes undergoing the Ex-PRESS shunt, a 26G needle was used to enter the anterior chamber about 1 mm from the limbus in a fashion parallel to the iris plane. The Ex-PRESS implant was then inserted through the site and the tip of the device was confirmed to be in the anterior chamber in the iris plane away from the cornea and without any iris obstruction. The scleral flap for both trabeculectomy and Ex-PRESS glaucoma shunt cases was then closed with four interrupted 10-0 nylon sutures to restrict flow to a "slow trickle" while the anterior chamber remains well maintained. The conjunctiva was closed in a water tight fashion in all cases using a running 9-0 nylon on a tapered needle. Prednisolone acetate was initiated the following day every 6 hours and tapered at the surgeon’s discretion postoperatively. Topical moxifloxacin was also initiated the following day every 6 hours for 7 to 10 days. Postoperative care was at the surgeon discretion but typically occurred on postoperative day 1, week 1, 4, and then about every 4 to 6 weeks for the first 3 months as needed. Laser suture lysis (LSL) was performed whenever needed to enhance flow (typically one suture at a time) and 5-FU (5-fluorouracil) injections were delivered postoperatively with a 30G needle superior to the bleb (volume 0.1 m of 5-FU 50 mg/mL = dose of 5 mg per injection) under topical proparacaine anesthetic when the surgeon needed to slow down the healing response as determined by extent of vascularity and degree of bleb encapsulation.

All patients had 1-year of follow-up data. Preoperative data collected included patient age, sex, race, glaucoma type, previous surgery, glaucoma medications, IOP, and visual acuity. Postoperative data included IOP, visual acuity, number of glaucoma medications, complications, and interventions, including LSL and 5-FU injections. Proportion of eyes achieving more than 30% IOP reduction from baseline without glaucoma medications was considered complete success. Proportion of eyes achieving more than 30% IOP reduction regardless of the number of glaucoma medications was considered qualified success. Data were collected at postoperative day (D) 1, week (W) 1, month (M) 1, M3, M6, M9, M12 year (Y) 1.

**Graph 1:**
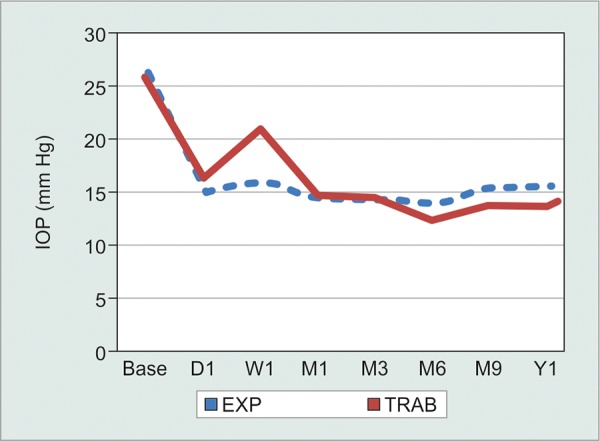
Average IOP over time

**Graph 2:**
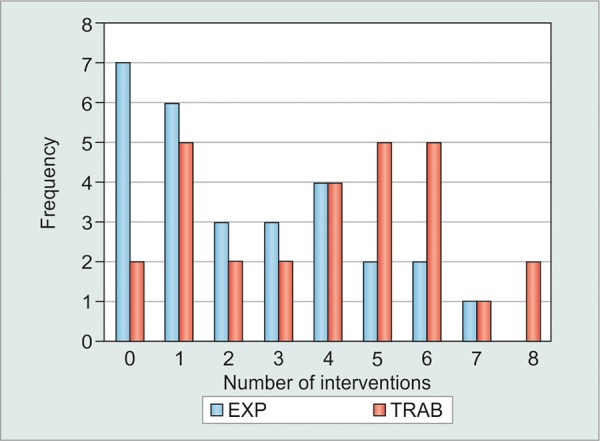
Cumulative interventions at month 3

**Graph 3:**
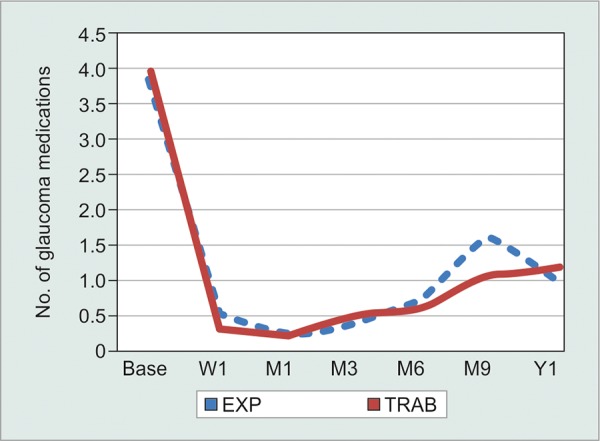
Average number of glaucoma medications over time

Means and standard deviation were calculated for all baseline parameters to ensure no significant differences, including age, IOP, visual acuity, and number of glaucoma medications. Simple unpaired t-tests were used to compare changes in IOP and number of glaucoma medications over time with baseline parameters. Simple unpaired t-tests were also used to compare IOP, number of glaucoma medications, and cumulative number of interventions (5-fluorouracil injections and LSL) between Ex-PRESS and trabeculectomy groups at respective time points. Fisher exact test was used to compare complication rates. Excel was used to construct graphs displayed in Graphs 1-3.

## RESULTS

The analysis included 56 subjects (E = 28, T = 28), the majority of whom had primary open-angle glaucoma. Baseline age, IOP, and number of glaucoma medications were similar between both groups (p > 0.05). There was a statistically significant reduction (p < 0.05) in IOP and number of glaucoma medications at all time points compared to baseline for both groups (Table 1). Extent of IOP reduction between groups was not statistically significant at any time point, except postoperative week 1 (lower in E group, Graph 1). The difference in mean number of glaucoma medications between groups was not significant except at 3 months (lower in E group, Graph 3).

As shown in Table 2 and Graph 2, the cumulative number of postoperative interventions within 3 months (LSL and 5-FU) was significantly greater in the TRAB than EXP (3.89 ± 2.4 *vs* 2.36 ± 2.2, p = 0.007). In addition, the cumulative number of 5-FU injections within 3 months was significantly greater in the TRAB than EXP group (2.25 ± 1.6 *vs* 1.25 ± 1.3, p = 0.007). Proportions of eyes achieving >30% IOP reduction from baseline, with and without medications, were comparable between both groups (Table 3). As for complications, they occurred in a total of eight patients in the trabeculectomy group and two patients in the Ex-PRESS group (28.5% and 0.01% respectively, p = 0.0776) and are summarized in Table 4. Notably, hypotony (IOP < 5 mm Hg) and bleb leak were the most common. Hypotony occurred only in the trab-eculectomy group (11%) with no statistical significance. One patient in the Ex-PRESS group had the iris blockin the shunt after 1 month following the operation, which required another revision surgery. Operations on three eyes failed in each of the E and T groups. Failure was defined as the need for re-filtration surgery.

**Table 1:** **Table 1:** Intraocular pressure and glaucoma medications at select time points

		*Baseline (E = 28,* *T = 28)*		*W1 (E = 28,* *T = 28)*		*M1 (E = 28,* *T = 28)*		*M3 (E = 28,* *T = 28)*		*M6 (E = 26,* *T = 20)*		*M12 (E = 14,* *T = 14)*	
Ex-PRESS IOP		26.1 ± 8.1		16.1 ± 10.5		14.6 ± 5.7		14.6 ± 5.8		14 ± 4.6		15.6 ± 7.4	
Trabeculectomy IOP		25.9 ± 9.3		21.1 ± 11.5		14.7 ± 7.6		14.5 ± 7.5		12.4 ± 4.8		13.7 ± 5.1	
p-value		0.47		0.047		0.48		0.33		0.12		0.15	
Ex-PRESS medications		3.82 ± 0.8		0.50 ± 1.0		0.25 ± 0.6		0.41 ± 0.7		0.76 ± 1.0		1.05 ± 1.4	
Trabeculectomy medications		3.93 ± 0.7		0.31 ± 0.7		0.23 ± 0.6		0.52 ± 0.9		0.62 ± 1.0		1.17 ± 1.4	
p-value		0.29		0.21		0.45		0.001		0.33		0.39	

W: Week; M: Month; p < 0.05 is considered statistically significant

**Table 2:** **Table 2:** Number of postoperative interventions

		*Exp*		*Trab*		*p-value*	
Total interventions (LSL, 5-FU)		2.36 ± 2.1		3.89 ± 2.4		0.007	
Cumulative LSL		1.14 ± 1.3		1.64 ± 1.2		0.07	
Cumulative 5-FU		1.25 ± 1.3		2.25 ± 1.6		0.007	

**Table 3:** **Table 3:** Success rates

		*Ex-PRESS* *(% eyes)*						*Trabeculectomy* *(% eyes)*							
		*M6*		*Y1*		*Y2*		*M6*		*Y1*		*Y2*		*X^2^*	
>30% no medication		50		36.4		28.6		63.4		37.5		35.7		0.98	
>30% medication/no medication		70.8		68.2		57.1		72.7		62.5		64.3		0.99	

**Table 4:** **Table 4:** Comparison of complication rates between trabeculectomy and Ex-PRESS groups

		*Ex-PRESS* *(n = 28)*		*Trabeculectomy* *(n = 28)*		*p-value*	
Hypotony		0		3		0.2364	
Choroidal detachment		1		1		1	
Hyphema		0		1		1	
Blebitis		0		1		1	
Bleb leak		1		2		1	
Total number of complications		2		8		0.0776	

## DISCUSSION

All glaucoma treatments aim to decrease IOP, which is the only modifiable factor affecting the optic nerve viability. During the last decade, the Ex-PRESS shunt emerged, aiming to provide an alternative surgical intervention with lower complication rates by eliminating intraop-erative iridectomy and sclerectomy and maintaining controlled aqueous drainage flow. However, any new procedure seeking to replace trabeculectomy should at least offer the same success rate but with less complica-tions.^8^ Therefore, many retrospective and prospective studies were conducted to study the efficacy of Ex-PRESS shunt and compare it to trabeculectomy.

Based on prevalence information from the Baltimore Eye Survey, the ratio of blacks to whites having glaucoma was four or higher.^9^ The advanced glaucoma intervention study (AGIS) showed that blacks were affected differently than whites by glaucoma interventions and that most glaucoma related outcomes were worse for blacks than whites.^10^ It is well-known that African origin patients experience an increased healing response with fibroblast and macrophage proliferation after surgery. This results in an exuberant healing response, causing contraction of the responsive scars at the site of surgery.^11-14^

Therefore, a change in surgical procedure for glaucoma in African origin patients can aim to decrease the rate of inflammation, and thus the filtration failure by avoiding extensive inflammatory and healing processes. To date, this is the first study comparing Ex-PRESS to trabeculectomy strictly in an African origin population.

In terms of IOP reduction from baseline measurement, there was a statistically significant reduction (p < 0.05) in both groups. The Ex-PRESS group had a baseline mean IOP of 26.1 ± 8.1 mm Hg and the trabeculectomy group had a baseline IOP of 25.9 ± 9.3 mm Hg and in both groups IOP was reduced to 15.7 ± 7.5 mm Hg and 16 ± 6.1 mm Hg respectively after 1 year. However, there was no statistical significance in the reduction of IOP between the two groups except at week 1, a result shared with different previous studies.

With respect to the number of glaucoma medications that were used to control IOP postoperatively, the number was reduced from 3.82 ± 0.8 and 3.93 ± 0.7 (p = 0.29) at baseline to 0.86 ± 1.00 and 1.93 ± 1.9 (p = 0.05) at 12 months for Ex-PRESS group and trabeculectomy group respectively, with no significant difference between the two groups except at 3 months and 2 years (lower in E group). In contrast, a similar study conducted by Marzette et al^15^ in 2011 comparing Ex-PRESS to trabeculectomy showed that the Ex-PRESS group had a small but significantly greater reduction in preoperative to postoperative medications compared with the trabeculectomy group (87 *vs* 81%, p = 0.023).

The term success in all studies varied slightly by definition, however, all discussed IOP ranges from 5 to 18 or 5 to 21 mm Hg with or without medication.^16^ In our study, a reduction of more than 30% with or without the use of medications showed comparable success rates between the two groups. Our results are similar to other studies done except for the two that showed higher complete success rates (using an IOP of 18 mm Hg as an upper limit) with Ex-PRESS implantation than with trabeculectomy.^8,17^

Suture lysis, 5-FU injection, or bleb needling are usually the most commonly reported additional interventions in most studies.^18^ There was a trend in the literature showing that less 5-FU injections were needed in the Ex-PRESS group but without any statistical signifi-cance.^8,15,19^ However, in our cohort, there was statistical significance in the difference of 5-FU injections needed postoperatively. Specifically, as shown in Table 2, the cumulative number of 5-FU injections given within the first 3 months was significantly lower in the Ex-PRESS group than in the trabeculectomy group (1.25 ± 1.3 *vs* 2.25 ± 1.6, p = 0.007). This result has not been indicated in other studies comparing Ex-PRESS to trabeculectomy. In fact, De Jong et al,^8^ Marzette et al,^15^ and Netland et al^19^ studies showed more needling with 5-FU in the T group but without any statistical significance. The decreased need for interventions early on proves two advantages. As African Americans have increased expression of TGF-(3 to stress, one can hypothesize that the Ex-PRESS group had less inflammation due to the lack of peripheral iridotomoy and sclerotomy and as such required less postoperative 5-FU to combat scarring. In addition, the decreasing interventions would remove potential sources of inflammation and failures.^20^ Furthermore, the decreased necessity of interventions can avoid the additional need for office visits and save time following glaucoma filtration surgery. As previously stated, the need for 5-FU injections is often required to treat robust postoperative inflammation and scar formation. Since our results demonstrate that the Ex-PRESS group required a significantly lower amount of 5-FU injections, this may indicate less postoperative inflammation in these patients.

Most of the studies comparing Ex-PRESS shunt to trabeculectomy showed similar rates and types of complications. In fact, only two studies, Maris et al^21^ and Marzette et al^15^ showed statistical significance in the difference between the Ex-PRESS and trabeculectomy groups regarding hypotony.^16^ In our cohort, all three cases of hypotony occurred early in the postoperative course (less than 3 months) and did not need any surgical intervention. Other complications in our study were also not significant or different from the literature.

This study has several limitations. These include its nonrandomized retrospective study design, a modest sample size, and the relatively short length of the follow-up time. The use of Ex-PRESS *vs* trabeculectomy was based on surgeon preference and, therefore, presents a possible bias.

## CONCLUSION

Our study is unique in that it was conducted in patients of African origin, and it showed a significantly lower requirement for postoperative 5-FU injections during the first 3 months following surgery in the Ex-PRESS group *vs* the trabeculectomy group. Consequently, this reduced the burden of care in a population where robust healing and scarring often require more intensive care postoperatively to ensure filtration success. In addition, there was no statistical difference between Ex-PRESS shunt and trabeculectomy in terms of IOP control, a number of medications needed postoperatively or impact on visual recovery. Hence, our findings are more or less consistent with other published reports showing that Ex-PRESS and trabeculectomy have a similar success rate. Our study provided similar evidence, however, in a very specific population, the African origin patients, who are at higher risks of glaucoma and have more pronounced inflammatory response leading to filtering bleb scarring. Therefore, the Ex-PRESS shunt could serve as an alternative filtration procedure to trabeculectomy in such a population. Further investigations in a prospective randomized study are warranted to reproduce the efficacy of Ex-PRESS shunt in African origin patients.

## CLINICAL SIGNIFICANCE

The possible benefit of this article is to help guiding ophthalmologists to select the type of glaucoma filtration surgery to undergo in an African American patient with glaucoma.
